# From Incidentaloma to Suspicion of Malignancy: The Diverse Clinical Presentation of Gonadal *Schistosomiasis mansoni*

**DOI:** 10.1155/2013/515910

**Published:** 2013-12-10

**Authors:** Laiana do Carmo Almeida, Marbele Guimarães de Oliveira, Fábio Meira Castro Pereira, José de Bessa Júnior

**Affiliations:** ^1^Department of Anatomic Pathology, School of Medicine, State University of Feira de Santana (UEFS), 44042330 Feira de Santana, BA, Brazil; ^2^Department of Urology, School of Medicine, State University of Feira de Santana (UEFS), 44042330 Feira de Santana, BA, Brazil

## Abstract

*Schistosomiasis* is the second most widespread parasitic disease in the world, second only to malaria. The usual places the *Schistosoma mansoni* can be found in are the rectal and sigmoidal venules, as well as other segments of the large intestine of men. It may also be present in other ectopic topographies. Gonadal schistosomiasis is an unusual presentation of *Schistosomiasis mansoni* and its different clinical signs and symptoms disrupt correct diagnosis and culminate in surgical treatment that is, in most cases, unnecessary. In this study, we report four cases of gonadal *Schistosomiasis mansoni*, two in the ovary and two in the testicles. These cases were clinically investigated as a bacterial infection, a benign neoplasm, and a suspected cancer, whilst one of them was an incidentaloma.

## 1. Introduction 

*Schistosomiasis* is the second most widespread parasitic disease in the world, second only to malaria, according to a study conducted by the World Health Organization (WHO). It is also an important cause of morbidity and significant mortality. Unusual presentations of *Schistosomiasis* have been described in many organs in the body. Usually these lesions are not clinically suspicious for *Schistosomiasis* and most of them are histologically diagnosed as incidental findings [1]. Testicular *Schistosomiasis* is rare, its diagnosis is difficult for mimicking testicular tumor, and it may cause unnecessary orchiectomy [2]. Ovarian *Schistosomiasis* has been described causing chronic granulomatous inflammation producing ovarian pseudotumor [3]. Gonadal *Schistosomiasis* is a rare presentation of ectopic *Schistosomiasis mansoni* both in testicles and ovary. We report four cases of gonadal *Schistosomiasis*, two in the ovary and two in the testicles. These were clinically investigated as a bacterial infection, a benign neoplasm, and a suspected cancer, while one of them was an incidentaloma.

## 2. Case Report

### 2.1. Case Presentation

**Figure 1:**
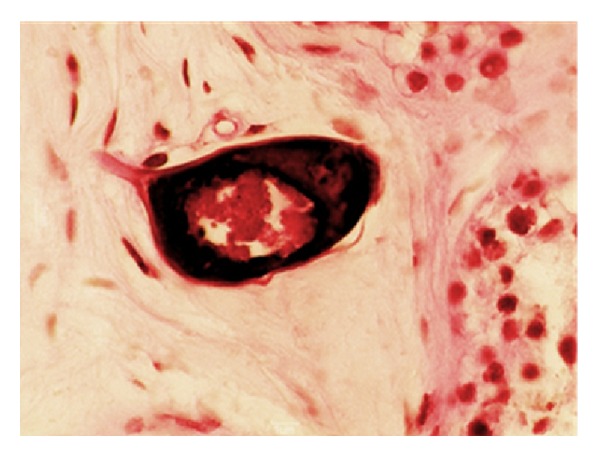
Histologic sections of the surgical specimen showed egg of *S. mansoni* with the characteristic lateral spine.

*Case 1: Testicular Schistosomiasis with No Clinical Suspicion*. An 84-year-old Brazilian man with a history of prostate cancer underwent bilateral orchiectomy for androgen-ablation therapy. In gross description, testicular skin color, consistency, and weight were usual. Histologic sections of the surgical specimen showed bilateral hypospermatogenesis and numerous calcified eggs of *S. mansoni* (Figure 1). Henceforth, this case has been presented as an incidentaloma in surgical specimen of bilateral orchiectomy for treatment of prostate cancer.

**Figure 2:**
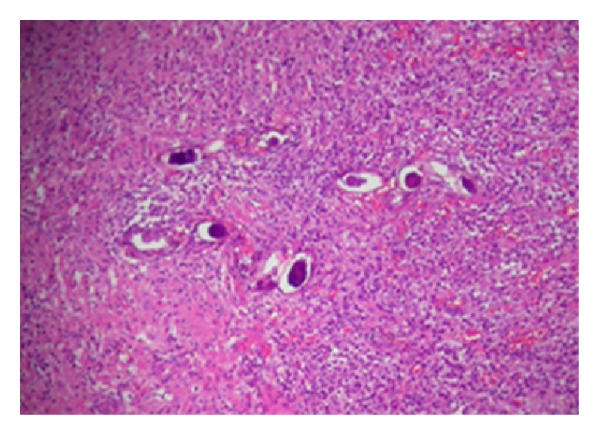
Histopathologically, ovarian parenchyma chronic inflammation involving numerous viable *S. mansoni* eggs often containing miracidium.

*Case 2: Ovarian Schistosomiasis with Suspected Bacterial Infection*. A 41-year-old Brazilian woman underwent oophorectomy because of pelvic pain and clinical suspicion of tubo-ovarian abscess. Macroscopically, we observed increased ovarian size measuring 6.5 × 5.5 × 3.0 cm with slightly lobed outer surface, elastic consistency, and brownish-yellow coloring. It consisted of six cystic cavities, the largest one measuring 2.3 × 1.5 cm with brownish walls and devoid of content. Histopathologically, cysts showed inner surface lined by granulation tissue, fibrin, and necrotic tissue; its wall was composed of extensive fibrosis and newly formed vessels associated with chronic granulomatous inflammation rich in eosinophils and neutrophils involving viable and nonviable *S. mansoni* eggs (Figure 2). Therefore, we have concluded that it consists of a case of *Schistosomiasis* with abscess and adjacent ovarian parenchyma with follicular cysts.

*Case 3: Ovarian Schistosomiasis with Suspected Benign Neoplasm*. A 44-year-old Brazilian woman with clinical and sonographic suspicion of dermoid cyst, salpingitis, and hydatid of Morgagni underwent left salpingo-oophorectomy and removal of cyst in the right uterine horn. Grossly, the left ovary was sectioned into two fragments which measured 3.4 × 2.0 × 2.5 cm and 2.0 × 1.0 × 0.7 cm. These fragments showed yellowish-brown coloration, elastic consistency, and lobulated surface. On sectioning, the tissue was brown and had an orange area with hemorrhagic aspect inside (suggestive of corpus luteum). The histological sections showed a cystic structure whose wall was lined by polygonal or polyhedral cells. Both cell types are filled with lipid droplets and have centrally located nuclei (compatible with corpus luteum). The adjacent ovarian tissue contained numerous calcified (nonviable) eggs of *S. mansoni*, surrounded by old granulomas. We conclude that it consists of a hemorrhagic corpus luteum associated with granulomatous chronic inflammation in cicatricial phase with calcified eggs of *S. mansoni*.

**Figure 3:**
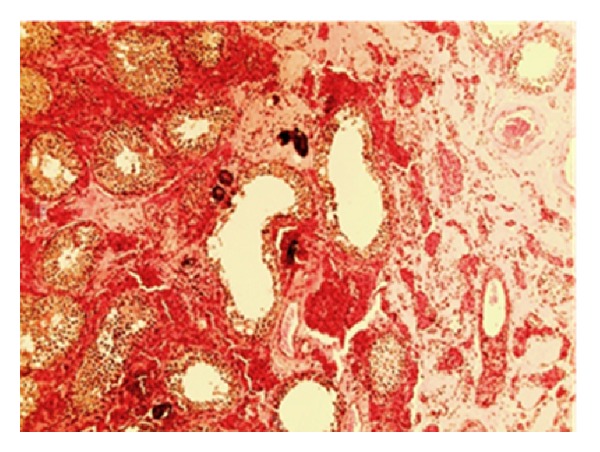
Testicular parenchyma with calcified *S. mansoni* eggs.

*Case 4: Testicular Schistosomiasis with Suspected Cancer*. A 40-year-old Brazilian man underwent left orchiectomy due to testicular mass suspicious of cancer. On examination of the surgical specimen, the testicle weighed 30 g and measured 2.5 × 1.8 cm in its greatest dimension. On gross description, he presented a heterogeneous parenchyma with areas of imprecise limits and alternating yellowish and brownish colors. Histopathologically, we noted numerous calcified *S. mansoni* eggs (Figure 3), which leads to the description of a testicular *Schistosomiasis* mimicking clinically a malignant neoplasm.

## 3. Discussion

Despite high endemicity of *Schistosomiasis* in various parts of the world, the finding of *S. mansoni* eggs in other organs is an unusual phenomenon, sometimes with serious clinical complications. Little attention has been given to the possible implications that these ectopic locations can ultimately have on understanding the behavior of the parasite [4]. The ectopic presentations occur when *S. mansoni* eggs or their adult form is found away from the portal system, as in the appendix, gallbladder, pancreas, peritoneum, urogenital tract, central nervous system, myocardium, skin, esophagus, stomach, thyroid, and adrenals [3]. Ectopic presentations can affect individuals of any parasitic load even after years of primary exposure [5].

The gonadal *Schistosomiasis* is extremely rare. In a recent review, we identified only 13 cases of testicular *Schistosomiasis*. Eight presented clinically as testicular edema, two with infertility and two with nodules. There was no predominance between right and left testicles [2]. The actual proportion of *Schistosomiasis* ovarian currently reported may not represent the actual rates of female genital involvement and reflect an underestimation of the disease [3].

Testicular *Schistosomiasis* is caused by migration of *S. mansoni* eggs through venous channels between the internal spermatic and mesenteric veins [4]. The route followed by the eggs is certainly the anastomosis between the inferior mesenteric vein and hemorrhoids, bladder, and pudendal plexus [6]. There are reports in the Brazilian literature which suggests that at the time of eggs laying, the female worms would drive to the terminal portion of the inferior mesenteric vein and superior hemorrhoidal plexus (branch of inferior mesenteric vein), thus reaching the walls of the vulva, cervix, and ovarian parenchyma [7]. However, the pathological mechanisms involved in ovarian infestation remain unknown and the diagnosis usually occurs retrospectively by histopathology [3].

Clinically, testicular *Schistosomiasis* may present as infertility, hydrocele, testicular growth, or testicular tumor [8]. The *S. mansoni* eggs can cause an allergic reaction in the testicles, which mimics testicular neoplasia [9]. On ultrasound, the characteristic lesion of testicular *Schistosomiasis* is a well-defined hypoechoic nodule with adjacent hyperechogenicity [10]. As this pattern is similar to that of hyperechoic testicular cancer, MRI can be better than ultrasound in showing intratesticular abnormalities [11]. The clinical features of ovarian *Schistosomiasis* are nonspecific and can mimic malignancy or infection in the female genital tract [12].

The scrotal involvement due to *Schistosomiasis* should be considered in the differential diagnosis of scrotal mass, especially in endemic areas [2]. As the differential diagnosis is difficult and simulates a cancer, it may lead to unnecessary orchiectomy [2]. Since no imaging method allows differentiation between benign and malignant disorders, biopsy is mandatory [13]. In endemic areas, this aberrant form of *Schistosomiasis* presentation should always be remembered, especially in patients with chronic, acute, or tumoral intrascrotal processes [6].

Ovarian *Schistosomiasis* must be considered as a possible diagnosis in women with menorrhagia who live in or have traveled to endemic areas. The improvement of diagnostic tools and monitoring with markers are important priorities to reduce morbidity from *Schistosomiasis* [5]. The gonadal *Schistosomiasis* treatment is performed with oxamniquine or praziquantel [2, 12]. In Brazil, the treatment is performed orally with praziquantel (50 mg/kg and 60 mg/kg body weight, single dose, for adults and children, resp.) and as second choice, oxamniquine (15 mg/kg and 20 mg/kg body weight, single dose, for adults and children, resp.), one hour after a meal [14].

## 4. Conclusions

We report four cases of gonadal *Schistosomiasis mansoni*, two in the ovary and two in the testicles. These cases were clinically investigated as a bacterial infection, a benign neoplasm, and a suspected cancer, as well as an incidentaloma. Gonadal *Schistosomiasis* is an unusual presentation of *Schistosomiasis mansoni* and its different clinical signs and symptoms disrupt correct diagnosis and culminate in surgical treatment that is in most cases unnecessary. In endemic areas, this etiologic possibility should be included in diagnostic research, because the standard treatment is pharmacological (oxamniquine or praziquantel).
